# Spatial–temporal evolution of soil gas Rn before two *Ms* ≥ 5.0 earthquakes in the mid-eastern of the Qilian fault zone (QLF)

**DOI:** 10.1038/s41598-023-46603-0

**Published:** 2023-12-06

**Authors:** Huiling Zhou, Yue Wan, Hejun Su, Chenhua Li

**Affiliations:** 1 The National Geophysics Observation Station, East Mountains West Road 450, Lanzhou, 730000 Gansu Province China; 2 China Earthquake Administration, Lanzhou Institute of Seismology, East Hills West Road 450, Lanzhou, 730000 China

**Keywords:** Natural hazards, Solid Earth sciences

## Abstract

The mid-eastern segment of the Qilianshan fault zone (QLF) on the northeastern margin of the Qinghai–Tibet Plateau is considered one of the key seismic hazard areas. The Zhangye *Ms*5.0 earthquake and Menyuan *Ms*6.9 earthquake are the two *Ms* ≥ 5.0 earthquakes in recent years. The spatio-temporal evolution of Rn across the fault before the two *Ms* ≥ 5.0 earthquakes were explored by combining a solid seismogenic model and numerical simulation results in this study. The results demonstrates the spatial distribution of Rn concentration intensity varies over time, indicating the evolving characteristics of fracture zone activity. The time-series variation characteristics are closely related the Zhangye *Ms*5.0 earthquake and Menyuan *Ms*6.9 earthquake. Overall, in the seismic source area and surrounding medium area of Zhangye *Ms*5.0 earthquake, the soil gas Rn anomaly across faults characterized by a turning upward trend after continuous decline. The closer to the source area, the more obvious the upward trend. For Menyuan *Ms*6.9 earthquake, the survey line (HT1) located in the main fracture zone of the earthquake and the survey line (HT7,30km from the epicenter) closer to the epicenter also showed a similar trend, while the other measurement lines in far-field exhibit declining trend before the Menyuan *Ms*6.9 earthquake. Therefore, the continuous decline trend of soil gas may be crucial information for medium-term earthquake preparation in the seismogenic zone, and the trend of turning upward after continuous decline is a significant signal of short-term seismogenic event in far-field. This research could improve the understanding of the anomalous features of soil gas precursors and tracking the active sections of the fault. According to the model, the earthquake area canseismic source area, the surrounding medium area be divided into three sections: the seismic source area, the surrounding medium area, and the fracture fragmentation area.

## Introduction

Subsurface fluids constitute essential components of the Earth system, playing a critical role in Earth’s evolution as an effective medium for interconnecting the planet's various layers^1–6^. Soil gas geochemical properties of deep gases discharged in geologically active areas has been extensively employed for precursors of tectonic (e.g., earthquakes and fault activity)^7–15^. The preparation and occurrence of earthquakes encompass a complex physical and chemical process, involving the energy transfer of deep materials, changes in medium conditions, and interactions between underground fluids, such as water and gas, and rocks under stress^13–17^. This process necessitates the migration, differentiation, and evolution of fluids^16–18^. Active fault zones serve as channels for underground fluid escape and are crucial locations for earthquake preparation and occurrence^16–24^. The gas geochemical properties of active fracture zones are often linked to the region's seismic activity^25–27^. In fact, these properties have been successfully utilized to elucidate key earthquake mechanisms along major active fault zones in Taiwan, Japan, and China^28–33^. Soil gases (CO_2_, CH_4_, Rn, Hg, etc.) in fracture zones frequently exhibit anomalies before and after earthquakes^21,34^, with the magnitude of these anomalies often fluctuating alongside seismic activity^35^. Research on the relationship between released CO_2_ fluxes and seismic activity in the L'Aquila region and the Apennines in Italy has demonstrated that CO_2_ gas released through rupture zones plays a crucial role in earthquake nucleation, occurrence, and aftershock activity^36^. Continuous monitoring of soil gas Rn concentrations on the Muzaffarabad fault in Pakistan abnormal Rn concentrations approximately 30 days prior to seven earthquakes, with magnitudes ranging from 0.8 to 4.9, impacting the rift^37^. Subsequent to the Wenchuan *Ms*8.0 earthquake, anomalies in soil gas He, CH_4_, Rn, H2, and Hg concentrations were found to be closely associated with surrounding aftershocks during drilling in the seismogenic rupture zone by scientific drilling holes No.1 (WFSD-1) and No. 2 (WFSD-2)^35,38,39^. Emission of CO_2_ was observed in association with earthquakes at the Lassen Peak volcano (Cascades Range, USA) (Ingebritsen et al., 2015) and the Eger Rift (Czech Republic), indicating close connections between the Earth's surface and its crust by fluid transport. 575 earthquakes occurred at Cava dei Selci (Colli Albani Volcano, Italy) from 2009 to 2021 showed that increase of CO_2_ flux seemed related to extensional deep earthquakes^40^. Additionally, the soil gas He and H_2_ anomalies in the Longmenshan rupture zone were observed to decrease as aftershock intensity diminished.

It is evident that underground fluids have gained increasing prominence in earthquake prediction and related studies in recent years. However, due to difficulty in natural earthquake prediction, research on combining earthquake monitoring results with physical prediction mechanisms is still very scarce. Most observations on seismic geochemical effects lack physical mechanism support for identifying anomalies. This is the key to break through the extraction of fault Soil gas earthquake anomaly precursors. The mid-eastern region of the Qilian Mountains Fault (QLF) is considered a key hazard area in Mainland China. Since 2016, six periods of Rn concentration data have been gathered from nine measurement lines deployed across the Middle East section of the QLF. On January 8, 2022, an *Ms*6.9 earthquake (37.77° N, 101.26° E) occurred in Menyuan County, Haibei Prefecture, Qinghai Province, China, with a focal depth of approximately 10 km. Additionally, the Zhangye *Ms*5.0 earthquake took place in the middle western section of the Qilian Mountain seismic belt on September 16, 2019. This study aims to investigate the spatial and temporal evolution of cross-fault Rn concentrations prior to two *Ms* ≥ 5.0 earthquakes in order to explore effective information on earthquake precursors. The research could enhance our understanding of the anomalous features of soil gas precursors and facilitate tracking of active fault sections.

## Geological setting

**Figure 1:**
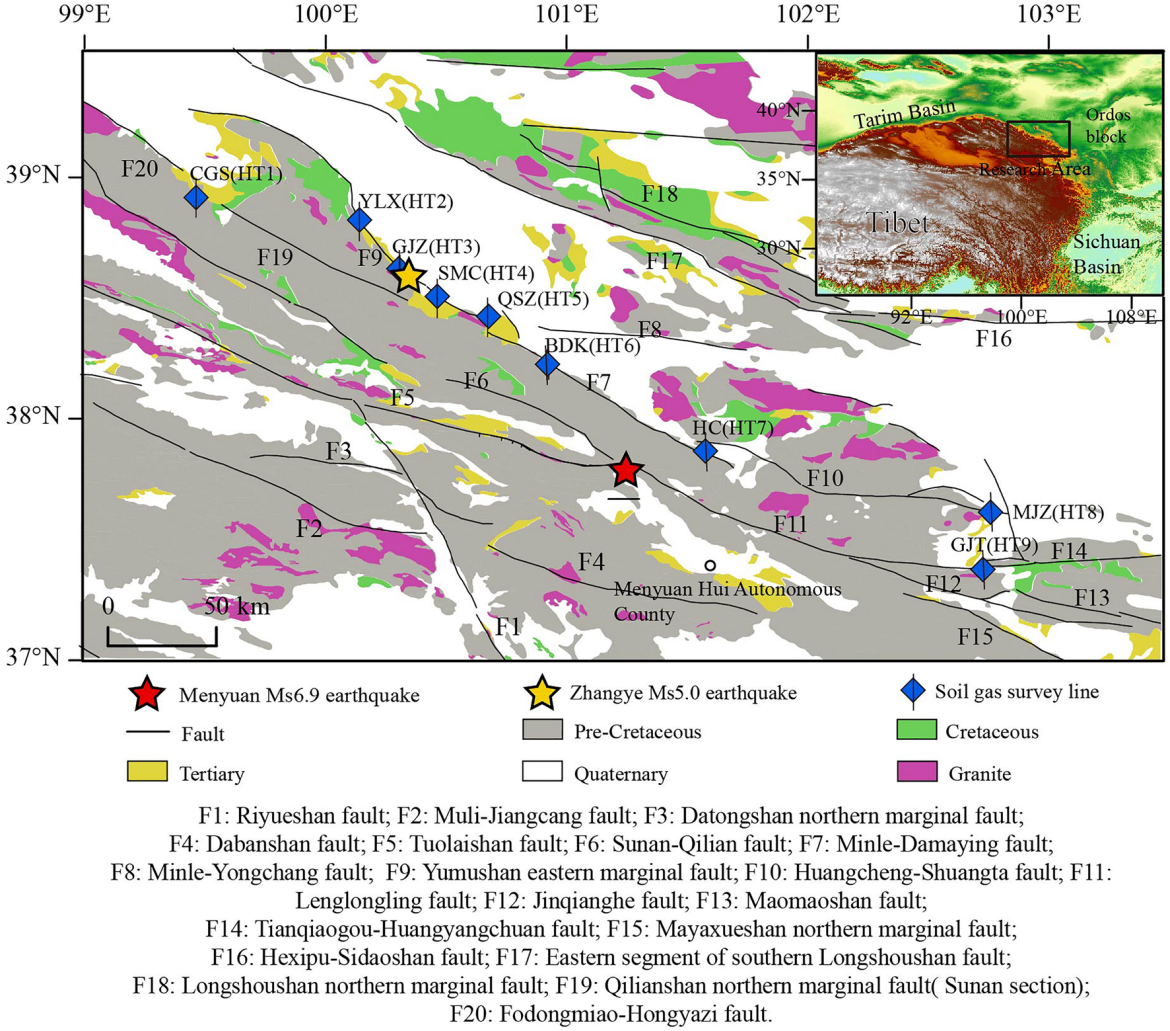
Tectonic settings of the middle-eastern section of the Qilian Mountains fault zone and the distribution of the soil gas measurement lines (Geology data set is provided by Geospatial Data Cloud site, Computer Network Information Center, Chinese Academy of Sciences (http://www.gscloud.cn); Active faults data are provided by sharing Infrastructure of National Earthquake Data Center (http://data.earthquake.cn)).

The central and eastern sections of the Qilian Mountains are predominantly located along the northeastern edge of the Tibetan Plateau, which is subject to the ongoing extrusion and collisional effects of the Indian and Eurasian plates from a distance^41,42^. This region is characterized by tectonic deformation and intersected by numerous active faults, rendering it one of the most seismically active and intense areas in mainland China, with substantial crustal movements^43–46^. The primary active fracture zones in this study area include the Sunan-Qilian fault, the Yumushan fault, the Minle-Damaying fault, the Huangcheng-Shuangta fault, and the Lenglongling fault, all of which are crucial components of the Qilian Mountains' northern margin (Fig. 1).These faults exhibit a NWW orientation and consist of multiple compression-torsional fractures arranged parallel to one another and in oblique rows^47^. The fault zone demonstrates a high degree of thrust characteristics, with the thrust fault cutting through strata from various periods, leading to the overthrusting of pre-Cenozoic strata, metamorphic rocks, and Paleozoic granites on Neogene mudstone and early-middle Pleistocene unconsolidated gravel. The zone also displays significant recent tectonic activity^48^. The Lenglongling fault, part of the North Qilian active fault zone, is situated at the northeastern boundary of the Tibetan Plateau uplift zone. Geotectonically, the fault is positioned within the North Qilian Fold Belt, north of the corridor transition zone and south of the Middle Qilian Uplift Zone^49^. The fault's eastern end connects to the Gulang fault and the Maomaoshan fault, while the western end links to the northern edge of the Tolaishan fault^50^. Approximately 120 km in length, the Lenglongling fault exhibits distinct linear characteristics^51–53^ and generally strikes at 110° N ~ 115° E. The fault's late Quaternary features include left-lateral strike-slip movement with a minor amount of dip-slip motion^54–57^. Although the Lenglongling fault's Quaternary slip rate remains contentious, it is still considered to have the highest slip rate within the Qilian-Haiyuan fault zone and plays a significant role in plateau deformation^58^.

## Date and methods

### Survey line layout and measuring instruments

**Figure 2:**
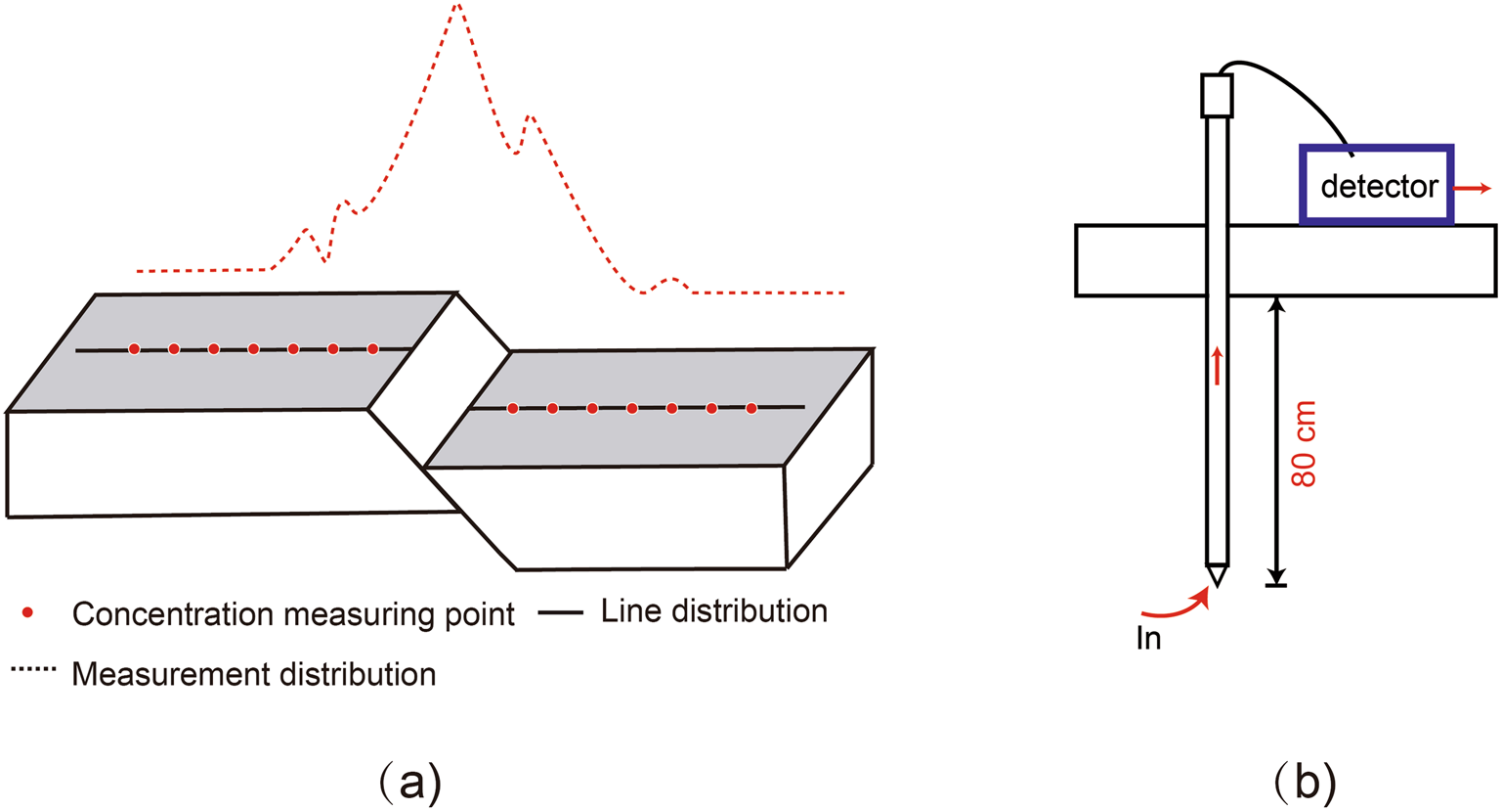
Schematic diagram of field sampling methods. (**a**) Laying of measurement lines; (**b**) Measurement Method.

Soil gas radon (Rn) measurements were conducted at nine survey sites (i.e., CGS, YLX, GJZ, SMC, QSZ, BDK, HC, MJZ, and GJT sites, numbered HT1 to HT9) situated on various NWW-oriented faults running parallel to each other and in oblique rows, covering the mid-eastern region of the Qilian fault (Table 1). The CGS (HT1) is located on the Sunan-Qilian fault, YLX (HT2) and GJT (HT3) are located on the Yumushan fault, SMC (HT4), QSZ (HT5), and BDK (HT6) are situated on the Minle-Damaying fault, HC (HT7) and MJZ (HT8) are on the Huangcheng-Shuangta fault, and GJT (HT9) is located on the Lenglongling fault. The survey line is oriented perpendicular to the fault strike, with 10-m intervals and 10–12 survey points arranged on each line to fully encompass the fault (Fig. 2).

**Table 1:** List of soil gas Rn survey line in the mid-eastern section of QLF.

Site	No	Longitude (°E)	Latitude (°N)	Number of measurement points	Length of survey line (m)	Direction of line measuring (°)	Fault zone
CGS	HT1	99.49	38.91	10	150	345	Sunan-Qilian fault
YLX	HT2	100.17	38.8	10	150	308	Yumushan fault
GJZ	HT3	100.33	38.61	10	150	25	Yumushan fault
SMC	HT4	100.48	38.5	10	150	200	Minle-Damaying fault
QSZ	HT5	100.7	38.41	10	150	50	Minle-Damaying fault
BDK	HT6	100.94	38.21	10	150	150	Minle-Damaying fault
HC	HT7	101.58	37.85	12	150	355	Huangcheng-Shuangta fault
MJZ	HT8	102.78	37.6	10	150	180	Huangcheng-Shuangta fault
GJT	HT9	102.74	37.37	10	150	45	Lenglongling fault

To minimize the impact of meteorology on soil gas concentrations, surveys were conducted under stable meteorological conditions in June of each year from 2016 to 2021. Rn concentrations, temperature, humidity, and atmospheric pressure were simultaneously measured using an AlphaGUARD detector (model PQ2000, Saphymo GmbH, Germany). According to the detector's requirements, 15 to 20 values were measured during each assessment. All samples were collected using a stainless-steel probe inserted into the ground at a depth of 80–100 cm, depending on soil consistency and thickness, in order to minimize the effects of meteorological variables^59–61^. No extreme climatic variations were observed during the monitoring period, and the meteorological conditions at each measuring point remained relatively stable.

### Data analysis method

In this paper,the concentration strength of each survey site was calculated.Concentration intensity refers to the measurement of the change degree of the of fault gas concentration near the fault plane relative to the background concentration. It can intuitively reflect several characteristics of fault media such as porosity, fragmentation, and fracture development, as well as the mechanics and motion state of fault activity It is the main characteristic parameter for analyzing the spatial characteristics of fault gas.The calculation formula is as follows:

Concentration strength (S) = Anomaly threshold/background value.

The background value is obtained by calculating the mean value of the remaining data after removing jump values, reflecting the normal accumulation level of specific elements or components in a given fault zone. The anomaly threshold can be expressed as the background value plus ‘n’ standard deviations (n = 1, 2, 3,…); in this case, n = 2 was used. The purpose of this is to eliminate the measured value instability caused by environment. Calculations and evaluations led to the decision to use the maximum value method for determining the concentration strengthof soil gas Rn(*S_*max). This approach utilizes the maximum abnormal concentration values to calculate concentration intensity, better explaining the peak value of the concentration intensity for each profile. Additionally, this method minimizes differences in site-effect factors that influence gas release concentrations, such as soil and rock properties^62,63^. Details of the nine survey lines are listed in Table 1.

## Results

### Soil temperature, humidity and atmospheric pressure

The average, maximum, and minimum values of soil temperature, humidity, and atmospheric pressure for each survey line are presented in Table 2. From 2016 to 2021, the soil temperature variation range for all measurement lines spanned from 11.7 °C to 35 °C. Soil temperatures were lower in the eastern section of the fracture zone measurement lines compared to the western section; however, the soil temperature variation remained more stable across six periods at each site. During this period, soil moisture variation from HT1 to HT9 ranged between 3.6 and 58.8. The humidity for all survey lines in 2018 was low, which is associated with that year's precipitation. The humidity differences in other years were minimal.As observed in Fig. 1s, the atmospheric pressure at each measurement line remained highly stable. Except for HT2, where the atmospheric pressure was concentrated around 820 mbar, the atmospheric pressure in the remaining measurement lines was approximately 800 mbar. This indicates that the measurement environment was relatively stable throughout the monitoring period.

**Table 2:** Results of the Soil temperature, soil humidity, atmospheric pressure of every survey line of QLF from 2016 to 2021(T: Temperature (°C); P: Pressure(m/bar); H: Humidity(%)).

NO		HT1	HT2	HT3	HT4	HT5	HT6	HT7	HT8	HT9
Site		CGS	YLX	GJZ	SMC	QSZ	BDK	HC	MJZ	GJT
16-Jun	T	22.4	20.8	28.9	29	21.1	21.9	20.6	26.4	23.2
	H	13.2	27.2	39.2	18.8	28	49.9	28.7	29.1	24.9
	P	799.6	819.9	798.5	799.5	801.2	803	801	801.8	756.8
17-Jun	T	22.6	29.8	23.9	33.5	26.7	22.1	20	27.6	24.7
	H	34	26.9	31.5	14.2	21.3	20	18.3	18.3	21.1
	P	801.5	802	799.6	798.2	799.1	800	800.3	803.6	800.6
18-Jun	T	27.2	29.8	33.7	32.3	25.9	12	13.3	32.4	17.5
	H	5.3	4.7	4.8	7	6.5	30.2	25.5	3.6	30.3
	P	798.9	820.3	797.5	797.9	799.2	802	801.7	801.8	800.9
19-Jun	T	14	30	24.3	32.3	22.4	22.2	11.7	30.4	20.3
	H	31.6	12.3	38.9	8.9	28.3	36.1	31.7	21.1	32.7
	P	801.7	819.1	799.5	797.8	799.8	799.9	802	800.7	800.3
20-Jun	T	21.3	31.5	26	27.1	24.7	21.6	23.7	26.2	24.9
	H	37.6	22	31.5	48.4	35	58.5	40.6	30	36.6
	P	800.3	823.3	799.6	799.6	800	800.5	799.9	802.9	799.6
21-Jun	T	24.6	25.3	35	24.2	19.1	22.7	28.1	24.7	26.5
	H	37.3	37	23.8	52.8	56.9	37.9	39.8	33.2	37.1
	P	799.9	821.4	797.8	800	800.9	800.4	799.1	801.8	799.2

### Rn concentration of every survey line

Table 3 presents the soil gas Rn measurement results for 9 profile from 2016 to 2021 in the eastern section of the QLF. In 2016, Rn concentrations were relatively high at HT7, HT8, and HT9, with values of 36 Bq/L, 34 Bq/L, and 39 Bq/L, respectively. Concentrations were lower at HT2, HT3, and HT4, with values of 15 Bq/L, 9 Bq/L, and 13 Bq/L, respectively. Rn concentrations in the other lines ranged between 21 Bq/L and 23 Bq/L. In 2017, Rn concentrations at HT7, HT8, and HT9 were also higher than those at other locations, with values of 40 Bq/L, 50 Bq/L, and 60 Bq/L, respectively. The Rn concentration at HT1 was the lowest,with values of 18 Bq/L. In 2018, the easternmost sites HT8 and HT9 had the highest Rn concentrations, with values of 55 Bq/L and 72 Bq/L, respectively, while the westernmost sites HT1-HT3 had the lowest , with values of 17 Bq/L, 19 Bq/L, and 16 Bq/L, respectively. In 2019, the easternmost site HT9 had the highest Rn concentration of 72 Bq/L, the westernmost site HT1 had the lowest concentration at 12 Bq/L, and HT3 also exhibited a relatively low concentration of 15 Bq/L. The concentration range for other measurement lines was 24–37 Bq/L. In 2020, the Rn concentrations at the easternmost sites HT8 and HT9 were relatively high, at 41 Bq/L and 50 Bq/L, while the concentrations for other measurement lines ranged from 20 Bq/L to 27 Bq/L. In 2021, Rn concentrations at HT8 and HT9 reached 37 Bq/L and 48 Bq/L, respectively, surpassing those at other measurement lines. Rn concentration varied over time but also demonstrated relative stability, indicating the reliability of the measurement data.

**Table 3:** Results of the soil gas Rn concentrations (*D*_max) and strength (*S*_max) of the mid-eastern section of QLF from 2016 to 2021.

NO	Site	2016		2017		2018		2019		2020		2021	
		*D_*max	*S_*max	*D_*max	*S_*max	*D_*max	*S_*max	*D_*max	*S_*max	*D_*max	*S_*max	*D_*max	*S_*max
HT1	CGS	21	4.22	18	2.1	17	1.82	12	2.56	23	1.84	30	2.17
HT2	YLX	15	2.47	22	1.93	19	2.11	24	2.4	20	2.19	23	1.89
HT3	GJZ	9	2.2	21	2.09	16	1.62	15	1.74	24	2.18	23	1.48
HT4	SMC	13	2.25	29	2.09	26	2.28	26	2.29	22	1.74	25	1.82
HT5	QSZ	21	5.42	28	2.58	23	1.76	29	1.96	27	1.88	30	1.88
HT6	BDK	23	3.28	31	2.63	34	2.11	27	2.36	27	2.1	31	1.81
HT7	HC	36	3.5	40	2.18	26	1.77	34	2.25	26	1.76	31	1.95
HT8	MJZ	34	3.03	40	2.18	55	2.71	27	2.65	41	2.99	37	1.96
HT9	GJT	39	3.73	60	3.25	72	3.18	72	3.31	50	2.05	48	1.97

### Spatial distribution of Rn concentration intensity

**Figure 3:**
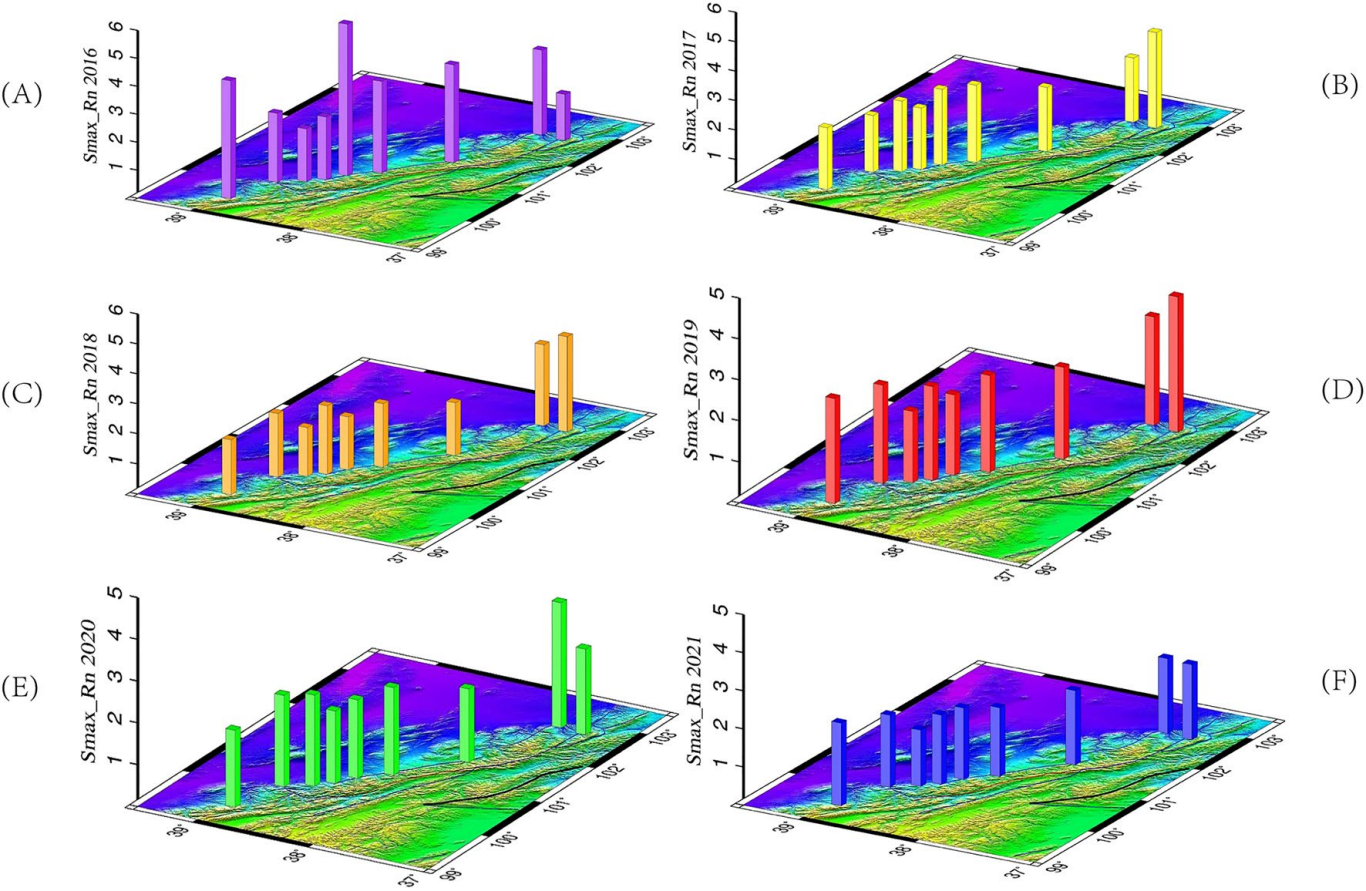
Spatial distribution of Rn gas concentration strength in cross-fault soils from 2016 to 2021 (Smax_Rn refers to Rn concentration strength; (**A**) (purple): 2016; (**B**) (yellow): 2017; (**C**) (ginger): 2018; (**D**) (red): 2019; (**E**) (green): 2020; (**F**) (blue): 2021).

The spatial distribution of Rn concentration intensity is depicted in Fig. 3. In 2016, the highest concentration intensity was observed at HT5 (5.42) in the middle section, followed by HT1(4.22) in the western section, and then HT9(3.73) and HT6 (3.28). The concentration intensities for HT2, HT3, HT4, and HT8 were 2.47, 2.2, 2.25, and 3.03, respectively. From 2017 to 2020, the spatial distribution characteristics of concentration intensity were stronger in the east than in the west, with the highest values observed at HT9 and HT8 in the eastern section. The HT9 values from 2017 to 2019 were 3.25, 3.18, and 3.31, respectively, while the HT8 value in 2020 was 2.99. Additionally, the western part of the QLF (Sunan section), including the Yumushan fault zone and the central part of the Minle Damaying fault zone, exhibited non-segmented Rn concentration intensity distributions between 1.74 and 2.63. In 2021, the spatial distribution was less distinct, with values across the entire fault zone uniformly ranging between 1.48 and 2.17. In conclusion, the spatial distribution of Rn concentration intensity varies over time, indicating the evolving characteristics of fracture zone activity.

The degree of fault gas release is influenced by subsurface medium conditions, local stress states, and both current and previous seismic activity^64–66^. The fault's permeability is crucial for controlling the intensity of soil gas concentration changes. Consequently, soil gas concentration strength is relatively higher in more open sections. Our previous research findings on the Haiyuan fault zone, the northern margin of the West Qinling fault, and the Liupanshan fault zone all support this perspective.

## Discussion

### Coupling relationship between fault soil Rn and fault activity in QLF

**Figure 4:**
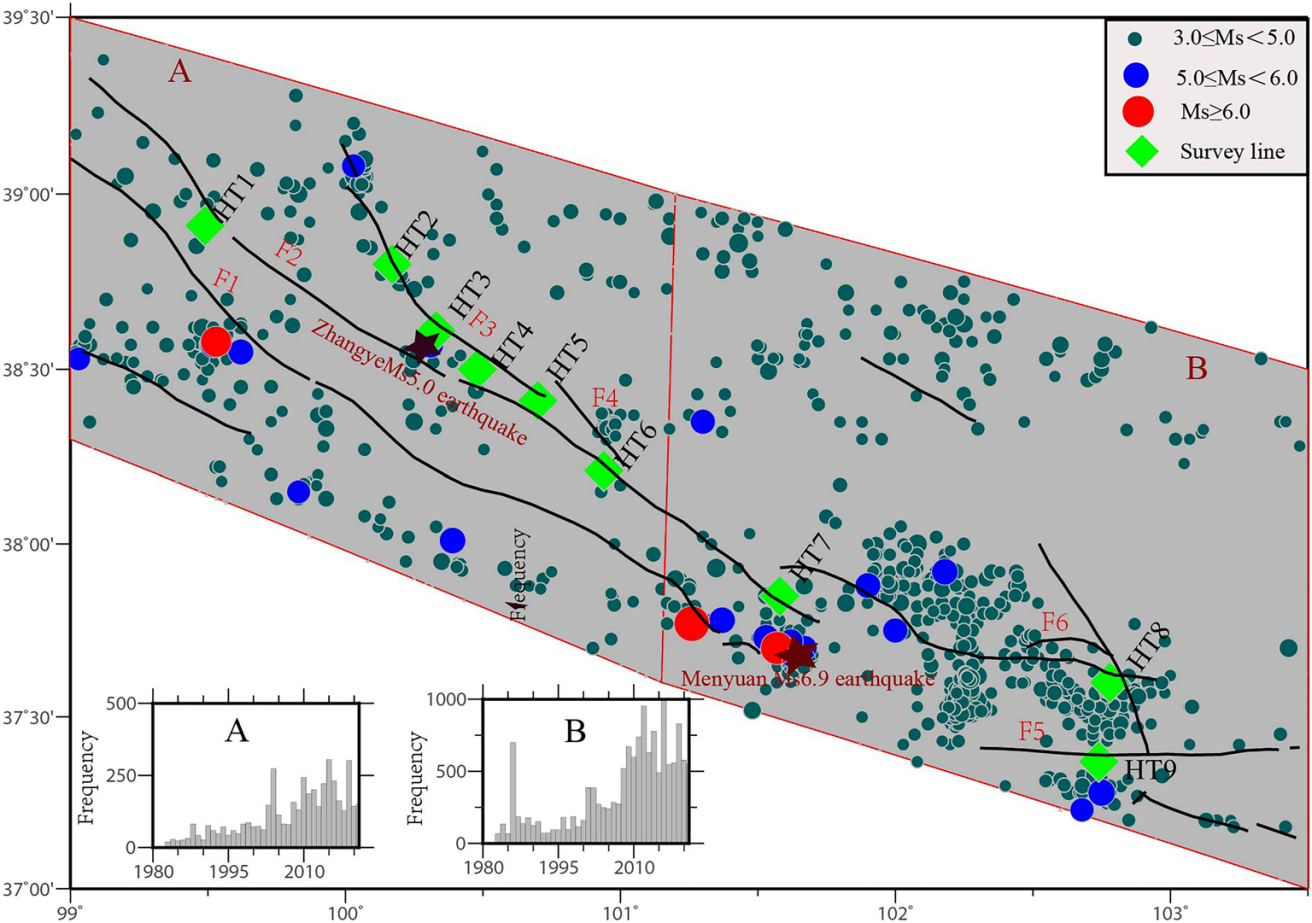
Distribution of seismic activity the mid-eastern of the Qilian fault zone (QLF) (F1: Sunan-Qilian Fault; F2: The northern edge of the Qilian Mountains, the southern section of Sunan; F3: Yumushan Fault; F4: Minle-Damaying Fault; F5: Lenglongling Fault; F6: Huangcheng-Shuangta Fault. Seismic data are provided by China Earthquake Networks Center (January 1971–April 2022)).

The spatial distribution of seismic activity is illustrated in Fig. 4. We divided the entire research area into two sections, east (A) and west (B).According to the time series statistics of seismicity frequency in the eastern and western sections, the seismicity in this region is very intensive, and there is a trend change of small amplitude enhancement in the time series. It can be seen that the seismicity of QLF has obvious segmentation that it is more frequent seismicity in the east section than that in the west section. The Huangcheng–Shuangta fault, the Lenglongling fault, and their intersection exhibit the strongest seismic activities. Notable events such as the *Ms*6.9 earthquake in 2022, the Menyuan *Ms*6.4 in 1986, and the *Ms*6.4 earthquake in 2016 all occurred in this area. Additionally, earthquakes with Ms ≥ 5.0 and smaller seismic activities are more concentrated in this region. Moreover, using remote sensing image interpretation, field geological survey, fault displacement measurement, and geomorphic surface age determination, the recoil sliding rate of typical dislocation points of the Yumushan fault was found to be (0.55 ± 0.15) mm/a, and the left-sliding rate was (0.95 ± 0.11) mm/a^25^. The linear fitting results of the vertical sliding rate of the Minle-Damaying fault were (0.91 ± 0.09) mm/a^41^. The Huangcheng–Shuangta fault experienced significant activity in the Holocene, most notably the Gulang *Ms*8.0 earthquake in 1927^43^. The Quaternary slip rate of the Lenglongling fault was determined to be (4.3 ± 0.7) mm/a by radiocarbon dating methods, while the slip rate since the late Holocene was (4.3 ± 0.36) mm/a^49^. The Huangcheng–Shuangta and Lenglongling fracture zones exhibit more activity than other fracture zones, indicating a relatively open fault. This corresponds well with the spatial distribution pattern of soil gas Rn in the QLF.

### Trend evolutionary characteristics of Rn related to Ms ≥ 5.0 earthquake in QLF

**Figure 5:**
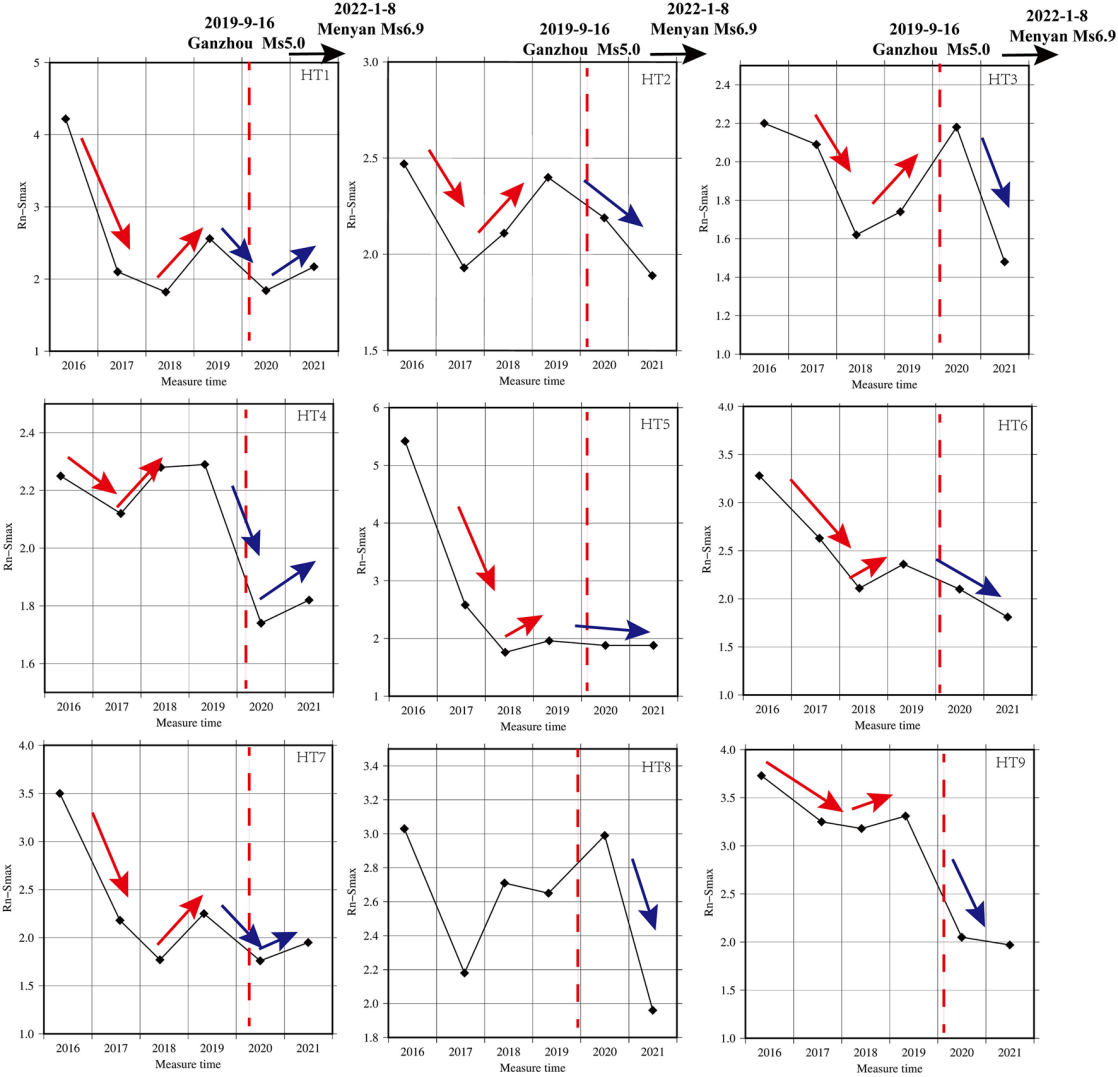
Radon concentration intensity time-series trend related to two Ms≥5.0 earthquakes in the mid-eastern of the Qilian fault zone (QLF) (red arrows indicate the strend related to Zhangye Ms5.0 earthquake (red dashed line) and blue arrows related to Menyuan Ms6.9 earthquake).

Since the initiation of cross-fault soil gas observations in the eastern QLF, the Menyuan *Ms*6.9 earthquake on January 8, 2022, and the Zhangye *Ms*5.0 earthquake on September 16, 2019 have occurred. The seismogenic fault for the Menyuan *Ms*6.9 earthquake is the western segment of the Lenglongling fault, which ruptured to the Tolashan fault on the west side of its northwest end^67–69^, while the Zhangye *Ms*5.0 seismogenic fault is the northern margin fault of the Qilian Mountain (Sunan Qilian section).The Rn trend changes before and after 2019 have captured our particular interest, as they may be related to the Zhangye *Ms*5.0 earthquake(Table 4). The Rn concentration intensity of all lines exhibited an apparent "V" pattern trend, with a significant decrease followed by an upward turn before 2019 (Fig. 5). However, differences in the range of change were observed. Specifically, HT1, located in the Sunan-Qilian fault and 80 km from the epicenter of the Zhangye *Ms*5.0 earthquake, displayed a significant decrease in Rn concentration intensity from 2016 to 2018, followed by an ascent before the earthquake. HT2 and HT3, situated in the Yumushan fault and 2–27 km from the epicenter of the Zhangye *Ms*5.0 earthquake, showed different patterns.The Rn concentration intensity of HT2 decreased significantly from 2016 to 2017 and increased significantly from 2017 to 2019, while the Rn concentration intensity of HT3 declined from 2016 to 2018 and increased significantly from 2018 to 2019. HT4-HT6, located in the Minle-Damaying fault with a distance of approximately 16–67 km from the epicenter, exhibited varying trends. Although HT4 was closest to the epicenter, the trend of continuous decline was not apparent, but an upward turn was observed before the Zhangye *Ms*5.0 earthquake. The trends of HT5 and HT6 showed a significant continuous decline followed by a slight upward turn before the earthquake.HT7 and HT8 are located in the Huangcheng-Shuangta fault. The Rn concentration intensity of HT8 increased after a continuous decline. However, the trend of HT9, which is 250 km away, was evidently weaker than that of other survey lines before the Zhangye *Ms*5.0 earthquake.

**Table 4:** Trend characteristics of Rn concentration intensity across the mid-eastern section of QLF from 2016 to 2021.

NO	Site	Trend characteristics related to the 2019 Zhangye Ms5.0 earthquake	Epicenter distance(km)	Trend characteristics related to the 2022 Menyuane Ms 6.9arthquake	Epicenter distance (km)	Fault zone
HT1	CGS	Descending from 2016 to 2018 and then increasing from 2018 to 2019	82	Descending from 2019 to 2020 and then increasing from 2020 to 2021	200	Sunan-Qilian fault
HT2	YLX	Descending from 2016 to 2017 and then increasing from 2017 to 2019	27	Continuous descending from 2019 to 2021	149	Yumushan fault
HT3	GJZ	Descending from 2016 to 2018 and then increasing from 2018 to 2019	2	Continuous descending from 2019 to 2021	124	Yumushan fault
HT4	SMC	Descending from 2016 to 2018 and then increasing from 2018 to 2019	16	Descending and then increasing	106	Minle-Damaying fault
HT5	QSZ	Descending from 2016 to 2018 and then increasing from 2018 to 2019	37	Continuous descending from 2019 to 2021	87	Minle-Damaying fault
HT6	BDK	Descending from 2016 to 2018 and then increasing from 2018 to 2019	67	Continuous descending from 2019 to 2021	56	Minle-Damaying fault
HT7	HC	Descending from 2016 to 2018 and then increasing from 2018 to 2019	136	Descending and then increasing	30	Huangcheng-Shuangta fault
HT8	MJZ	Descending from 2016 to 2017 and then increasing from 2017 to 2019	240	Continuous descending from 2019 to 2021	135	Huangcheng-Shuangta fault
HT9	GJT	Descending from 2016 to 2018 and then increasing from 2018 to 2019	250.43	Continuous descending from 2019 to 2021	138	Lenglongling fault

In addition, we observed that the Rn concentration intensity exhibited an ascending trend in 2020, following a declining background in HT1, HT4, and HT7 (Fig. 5 and Table 4). Specifically, these lines showed a decline from 2019 to 2020 and then an ascent from 2020 to 2021, forming a noticeable "V" pattern, with an upward turn following the decline. HT1 is situated in the primary rupture zone of the northern edge of the Qilian Mountains, where several *Ms* ≥ 3.0 events occurred after the Menyuan *Ms*6.9 earthquake. HT7 is located within the Huangcheng–Shuangta fault, approximately 30 km from the Menyuan *Ms*6.9 earthquake's epicenter. HT4 is positioned in the Minle–Damaying fault to the west of the Menyuan *Ms*6.9 earthquake, with an epicenter roughly 100 km away. Furthermore, with the exception of these three lines, all other measurement lines continued to exhibit a declining trend before the Menyuan *Ms*6.9 earthquake. We assumed that these trend changes may be related to the Menyuan *Ms*6.9 earthquake that occurred on January 8, 2022.

In summary, we found that Rn concentrations near the epicenter areas displayed an upward trend following a continuous decline before both *Ms* ≥ 5.0 earthquakes in QLF (Table 4 and Fig. 5). The persistent decline trend may be a crucial signal for moderate to strong earthquakes in the medium to long term. We have observed such trend characteristics before several moderately strong earthquakes on the northeastern margin of the Qinghai Tibet Plateau.

### The mechanism of Rn spatial–temporal evolution before Ms ≥ 5.0 earthquakes

**Figure 6:**
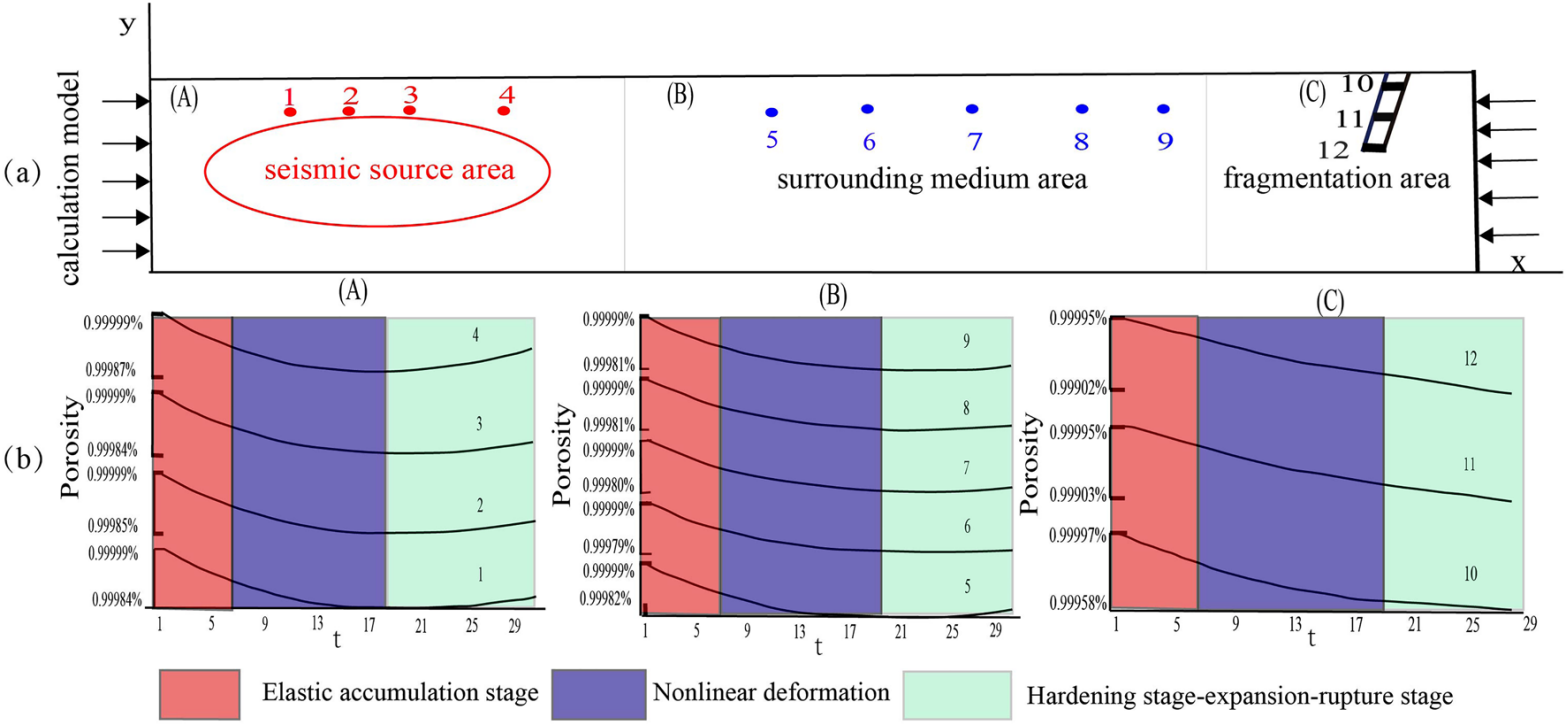
Solid seismogenic model and schematic diagram of numerical model ((**a**) is schematic of numerical simulation calculation model, (**b**) is the variation of porosity with time at various representative points in different regions) t represents different stages of earthquake preparation^75^.

Earthquake precursors are phenomena that manifest at specific stages of earthquake preparation. It is essential to connect the spatial–temporal evolution of precursors with the earthquake process and mechanism. Since the 1970s, the Fracture Collusion model (IPE) and Dilatancy Hypothesis model (DD) have been proposed. However, these models mainly address the issue of epicenter precursors, and they cannot explain the complex spatiotemporal evolution process of precursors^70^. Through extensive research on large earthquakes (*Ms* ≥ 6.0), it has been observed that strong earthquakes typically do not occur in the high seismicity zones of fault systems, but rather in seismic gaps^71,72^. Subsequently, the concept of seismic gaps was introduced, and a solid seismogenic model was established to elucidate the conditions, processes, and associated precursors of strong earthquakes, as well as their spatial–temporal evolution^73,74^. This model is supported by a wealth of evidence from deep structures, mechanical analyses, rock fracture experiments, numerical simulation results, and observed facts^75^. According to the model, the earthquake area can be divided into three sections: the seismic source area, the surrounding medium area, and the fracture fragmentation area (Fig. 6a). The source area may comprise a block with a relatively intact macroscopic rupture or a locked segment within a fault zone.The earthquake preparation process consists of several stages, including the elastic accumulation stage, the nonlinear deformation and local hardening stage, the local hardening stage and expansion, the large-scale expansion stage, and the rupture stage^76,77^. The seismogenic medium is a saturated two-phase medium composed of solid lithosphere and pore fluid. Under the influence of regional tectonic stress, the medium undergoes various stages of deformation, causing changes in the physical parameters of the rock^78,79^. Among these parameters, porosity is a significant quantity closely related to radon emission. As the rock's porosity increases, so does its surface area, radon ejection coefficient, and radon content in the rock's pore fluid. According to the solid seismogenic model, during the elastic accumulation stage and nonlinear deformation and local hardening stage in the seismic source area, porosity decreases due to compression^73,75,76^. Consequently, the soil gas radon anomaly exhibits a decreasing trend at the beginning and middle period of earthquake inception. However, during the local hardening stage, expansion, and large-scale expansion stage, porosity increases. Therefore, in the short-to medium-term of earthquake preparation, especially during the impending stage, the soil gas radon anomaly primarily trends upward. Furthermore, underground fluid anomalies first appear in the source area and surrounding medium area due to the earliest expansion occurring in the source region. During the medium to short-term seismogenic stage, the anomalous area expands, and numerous precursors emerge in the fracture fragmentation area, while the development of anomalies in the source area remains relatively stable. This phenomenon was confirmed by numerical simulation of the spatiotemporal evolution of underground fluid anomalies under the strong solid seismogenic model (Fig. 6b). Overall, in the seismic source area and surrounding medium area, the soil gas Rn anomaly across faults may exhibit a "V" shaped change trend, characterized by a turning upward trend after continuous decline^75^. The closer to the seismic source area, the earlier the turning upward time.Therefore, the continuous decline trend of soil gas may be crucial information for medium to long-term earthquake preparation, and the trend of turning upward after continuous decline is a significant signal of a medium and short-term seismogenic event. These important signals require a comprehensive understanding of the tectonic background and activity of fault zones.

This model effectively explains the relationship between two Ms ≥ 5.0 earthquakes in the QLF. For the Zhangye *Ms*5.0 earthquake, the Rn concentration intensity of HT2 (27km away from the epicenter) decreased significantly from 2016 to 2017 and increased significantly from 2017 to 2019. In HT3 (2 km away from the epicenter), the Rn concentration intensity decreased from 2016 to 2018 and increased significantly from 2018 to 2019. Other survey lines also demonstrated some trend anomalies, but the short-term anomalies (turning upward trend after continuous decline) were not more pronounced than in HT2 and HT3. For the Menyuan *Ms*6.9 earthquake, HT1, HT4, and HT7 also displayed turning upward trends after continuous decline before the earthquake. HT1 is located on the the northern edge of the Qilian Mountains, the southern section of Sunan, 200 km from the epicenter. Despite this distance, it is situated along the main fault at the northern margin of the Qilian fault. The Lenglongling fault zone, where the Menyuan *Ms*6.9 earthquake occurred, is also an integral component of the northern Qilian fold belt in terms of geotectonics. Several aftershocks with a magnitude of *Ms* ≥ 3.0 were recorded in the region following the Menyuan *Ms*6.9 earthquake. Furthermore, except for three lines, all other measurement lines exhibited a continuing decline trend before the Menyuan *Ms*6.9 earthquake. The Menyuan *Ms*6.9 seismic activity and tectonic deformation directly expose the adjustment and transmission of tectonic deformation throughout the Qilian Mountain fault zone. As the survey lines did not directly cross the seismic fault of the Menyuan *Ms*6.9 earthquake, the abnormal features were not as pronounced as those observed in the Zhangye *Ms*5.0 earthquake.

The response of soil gas Rn to seismic events is not only related to the magnitude and distance from the epicenter but also controlled by the tectonic stress of deep major faults. Thus, the present observation results and the analysis of seismic cases support the body seismogenic model and numerical simulation computation results. This model elucidates the relationship between tectonic geochemical characteristics and rupture blockage, offering theoretical support for determining the time, space, and intensity of seismogenesis. The robust body seismogenic model can clearly explain the relationship between tectonic geochemical features and fracture locking, providing theoretical support for estimating the time, space, and intensity of earthquake nucleation.

## Conclusion

In this study, we explored the spatial–temporal evolution characteristics of soil gas Rn from 2016 to 2021 in the mid-eastern of the Qilian fault zone (QLF) before Zhangye Ms5.0 earthquake and Menyuan Ms6.9 earthquake by combining a solid seismogenic model and numerical simulation results. By analyzing the spatial–temporal evolution characteristics of soil gas Rn before two Ms ≥ 5.0 earthquakes in the Qilian fault zone, the following conclusions can be drawn:

1. From a spatial distribution perspective, in 2016, the highest concentration intensity was observed in the middle (HT5) and west section (HT1). From 2017 to 2020, the spatial distribution characteristics of concentration intensity were stronger in the east than in the west.In 2021, the spatial distribution was less distinct, with values across the entire fault zone uniformly. The spatial distribution of Rn concentration intensity varies over time, indicating the evolving characteristics of fracture zone activity.
2. From a temporal evolution trend before earthquake,the soil gas Rn concentration intensity across mid-eastern of the Qilian fault zone(QLF) exhibited a significant response to the Zhangye *Ms*5.0 and Menyuan *Ms*6.9 earthquakes. For the Zhangye *Ms*5.0 earthquake, the Rn concentration intensity in major survey lines (except HT8 and HT9) showed a noticeable "V" temporal evolution trend, with an continue decline and then turn upward. For the Menyuan *Ms*6.9 earthquake, HT1 (located on the the northern edge of the Qilian Mountains) and HT4, and HT7() also displayed turning upward trends after continuous decline before the earthquake.While the other measurement lines exhibit a continued declining trend before the Menyuan Ms6.9 earthquake.
3. These observational facts were supported by the sturdy body seismogenic model and numerical simulation results. Consequently, we hypothesized that the continuous decline trend of fault gas may serve as a reliable indicator of the interlocked section of fault tectonic activity, and the trend of turning upward after continuous decline may represent a significant signal of a medium and short-term seismogenic event in the source area. Furthermore, the sturdy body seismogenic model could potentially provide theoretical support for determining the time, space, and intensity of seismogenesis based on the relationship between the tectonic geochemical characteristics and rupture locking.

In summary, seismic prediction remains a difficult global scientific problem, and fluid geochemistry is one of the potential tools for earthquake prediction. The extraction of earthquake precursor information from soil gas must be based on a specific physical mechanism of seismogenesis. The results of long-term research have shown that, by studying the physical mechanisms of forecasting, the sensitivity and convenience of fault gas can be used to trace locked segments directly on dangerous faults for the identification and determination of precursor information, which can compensate for the limitations of springs and well-exposure locations used by subsurface fluid.

## Data Availability

The data are available from the corresponding author upon request after publication of this paper.
